# Evaluating Eight Retrieval-Augmented Generation (RAG) Large Language Models’ Responses to Clinical Questions: A Comparative Study

**DOI:** 10.64898/2026.08.10.26360108

**Published:** 2026-08-12

**Authors:** Poppy A. Krump, Mallory N. Blasingame, Taneya Y. Koonce, Annette M. Williams, Jing Su, Nunzia B. Giuse

## Abstract

**Background:**

Large language models (LLMs) that use retrieval-augmented generation (RAG) are increasingly used to answer clinical questions, although the evaluation of these systems remains limited. Building on previous studies conducted by our team, this case report aimed to improve upon this knowledge gap by applying a reusable methodology to compare the performance of eight LLMs that utilize RAG techniques for evidence synthesis.

**Case Presentation:**

Eight commercially available RAG LLM tools (OpenEvidence, Undermind, Consensus, SciSpace, Elicit, MediSearch, EvidenceHunt, and Scite) were evaluated using twelve ChatGPT-generated clinical questions on the topics of treatment, etiology, and prognosis. To enable comparison, we prompted ChatGPT to identify all key unique medical concepts from the full set of LLM responses to each question. Concepts were categorized as critical (“must-have”) or non-critical (“nice-to-have”) for answering the clinical question. Experienced information scientists were consulted at each step for their expertise. Descriptive statistics and Kruskal-Wallis tests were used to compare performance across tools and question categories. No significant differences were found among the eight RAG LLMs in their coverage of “must-have” (p=0.95) or “nice-to-have” (p=0.16) key unique medical concepts, and no single tool consistently captured all identified concepts.

**Conclusions:**

These findings suggest that RAG LLMs may be supplementary tools for evidence retrieval and synthesis but cannot, at this time, fully replace comprehensive expert review of the medical literature. The evaluation framework presented here may be a useful model for future comparative assessments of rapidly evolving AI evidence synthesis tools.

## Full Text Availability

The license terms selected by the author(s) for this preprint version do not permit archiving in PMC. The full text is available from the preprint server.

